# Clinical characteristics, environmental factors and quality of life of children with atopic dermatitis: A single-centre cross-sectional study from South Africa

**DOI:** 10.4102/curationis.v49i1.2809

**Published:** 2026-10-20

**Authors:** Simangaliso Madolo, Mirabel K. Nanjoh, Kolawole D. Ogundeji

**Affiliations:** 1 Department of Public Health, Faculty of Medicine and Health Sciences, Walter Sisulu University, Mthatha, South Africa

**Keywords:** clinical characteristics, environmental factors, quality of life, children, atopic dermatitis, rural setting

## Abstract

**Background:**

Atopic dermatitis (AD) is a chronic inflammatory skin condition affecting children worldwide. Limited research exists on AD in rural settings.

**Objectives:**

This study aimed to investigate the clinical characteristics, environmental factors and quality of life of children with AD.

**Method:**

This is a cross-sectional research design. Children (*n* = 114) with AD were included in the survey, with parents serving as proxy informants. A semi-structured questionnaire and the validated Children’s Dermatology Life Quality Index (CDLQI) were used to collect data. Data were analysed using SPSS version 29. Descriptive statistics summarised demographics, clinical characteristics, environmental factors and the impact of AD on children’s quality of life. The Kruskal–Wallis test was used to analyse factors associated with the quality of life of children with AD at the 0.05 significance level.

**Results:**

The median age of the children was 6 years (interquartile range [IQR]: 4–9 years), with a high prevalence of mild AD (65%). The most affected body parts are the face (33.3%), the hands (23.7%) and the feet (20.2%). Key environmental triggers included pet ownership (81.6%), use of regular soap (98.2%) and seasonal exacerbation during winter (53.9%). Atopic dermatitis had a weak effect on the quality of life of most children (65.2%). The effect of AD on the quality of life was not influenced by gender, age group or the body part affected (*p* > 0.05).

**Conclusion:**

The quality of life of children in rural settings is primarily impacted by AD in areas such as schooling, sleeping and treatment domain.

**Contribution:**

Public health efforts should be scaled up to mitigate the burden of atopic dermatitis.

## Introduction

Atopic dermatitis (AD) is a common dermatosis in children with increasing prevalence worldwide (Hadi et al. 2021; Jiang & Sun 2018; Kelbore et al. 2024; Li et al. 2018). It is an inflammatory skin disease caused by the interaction of genetic and environmental factors (Da Silva Tenorio et al. 2022; Li et al. 2018; Seraphim et al. 2022; Wang et al. 2025; Wang & Ma 2019). It can affect any area of the body, especially the face, hand, knee and scalp, and the morphology depends on the age of onset. It is common and severe in children with a wide range of clinical manifestations, from mild to severe symptoms, impacting their daily lives (Xie et al. 2019).

Atopic dermatitis affects children’s health-related quality of life, increasing the burden on family finances and the healthcare system (Anderson et al. 2021; Neri et al. 2023). The disease symptoms could be uncontrollable in children, while sometimes the children live an everyday life, suggesting a complex nature of the condition. The acute stage is often characterised by severe pruritus, with children continuing to scratch and wanting to pierce the skin (Grant et al. 2019; Kelbore et al. 2024). At this period, the parents become uncomfortable and could try various remedies to soothe the itching. Literature is replete with the psychological, emotional and social effects of chronic AD on the child and family well-being (Na, Chung & Simpson 2019). Some studies reported on the loss of attendance at school or day care (Koszorú et al. 2019; Meltzer et al. 2020; Na et al. 2019; Sur et al. 2020).

According to Serrano et al. (2019), atopic dermatitis often starts during infancy in about 45% of cases and preschool to school-age in about 70% of cases. Environmental factors play a significant role in exacerbating atopic dermatitis in children: Exposure to pollutants, allergens, certain foods and microbes has been implicated (Hachmeriyan et al. 2025; Moran 2023; Moran et al. 2023). Also, there is growing concern about other factors such as climate, exposure to pets, breastfeeding, diet and allergens which can trigger the development of atopic dermatitis (Bonamonte et al. 2019). Based on the foregoing triggers, the clinical management of atopic dermatitis among children involves a multidisciplinary approach to identify and treat the environmental triggers. The treatment regimen includes emollients, topical corticosteroids, and, in severe cases, ultraviolet phototherapy.

However, despite the high prevalence and severity of atopic dermatitis among preschoolers and school-age children (3 to 11 years old) in Eastern Cape province, South Africa, there is limited understanding of the clinical characteristics and specific environmental factors that contribute to its development and exacerbation in this population. Most previous studies focused on genetic and immune system factors, while environmental factors such as climate and temperature, air particulate matter, exposure to allergens and irritants, dietary and nutritional deficiencies, socioeconomic status and healthcare factors remain poorly understood. This knowledge gap hinders the development of effective prevention and intervention strategies, thereby imposing a significant burden on families, the healthcare system and the economy. Therefore, this study aimed to address knowledge gaps by investigating clinical characteristics and environmental factors associated with the development of atopic dermatitis and its impact on children’s quality of life in the Eastern Cape Province, South Africa.

## Research methods and design

### Study design

A cross-sectional research design was used to identify the clinical characteristics and environmental factors associated with the development of atopic dermatitis and its impact on children’s quality of life in Mthatha, Eastern Cape Province, South Africa.

### Study setting

This study was conducted at the Nelson Mandela Academic Hospital dermatology clinic in Mthatha, Eastern Cape. Mthatha is a mid-sized town in the Eastern Cape.

### Study population

The study population was children with atopic dermatitis residing in Mthatha, Eastern Cape Province, and attending the Nelson Mandela Academic Hospital dermatology clinic.

### Sample size calculation

The sample size was calculated using the EpiDATA sample size calculator. The prevalence of atopic dermatitis in South Africa was 13.3% (Todd 2014). A precision of 7% and a confidence level of 95% were obtained. A sample size of 95 was obtained. To reduce the effect of missing data on the sample, an additional 19 (20% of 95) were added. The study included 114 respondents.

### Sampling method

The study used convenience sampling. The respondents were the parents of children who attended the dermatology clinic at Nelson Mandela Academic Hospital within the study period. The clinic opens 4 days weekly – Monday, Tuesday, Thursday and Friday. The data collection spans from 16 September 2024 to 21 November 2024.

Inclusion criteria:

- Children aged between 3 and 11 years.
- Residing in Mthatha, Eastern Cape province.
- Children diagnosed with atopic dermatitis.

The exclusion criteria are as follows:

- Children with other chronic skin conditions.
- Children with immunodeficiency disorders or receiving immunosuppressive therapy.
- Children who are not diagnosed with atopic dermatitis.

### Data collection instrument

The instruments deployed were a semi-structured questionnaire and the validated Children’s Dermatology Life Quality Index (CDLQI) scale. The questionnaire elicited information on the children’s demographics and clinical characteristics of patients with atopic dermatitis. body part affected by atopic dermatitis, the severity of the child’s atopic dermatitis and the environmental factors associated with atopic dermatitis. The CDLQI measured the impact of atopic dermatitis on children’s quality of life. The instruments were translated into isiXhosa, the Eastern Cape, South Africa’s indigenous language.

### Validity and reliability of the instrument

The semi-structured questionnaire was subjected to face, construct and content validity. The instrument was assessed by an expert in dermatology, Public Health and Biostatistics. Feedback from the experts’ assessment was considered, leading to modifications to some parts of the questionnaire. Also, a meeting was held with the researchers to collect data accurately based on the set inclusion and exclusion criteria.

The questionnaire was also pretested before the data collection commenced. A sample of 10 questionnaires was given to parents of children aged 3–11 years at Mthatha Regional Referral Hospital, and the same number of questionnaires were readministered 2 weeks later at the same hospital. The internal consistency of the items of the instrument was analysed with a coefficient of reliability of 0.848.

### Data collection procedure

During the clinic visit, a semi-structured questionnaire and the CDLQI were used to collect data from parents of children with atopic dermatitis. The data collection focused on sociodemographic, clinical and environmental factors associated with the development, recurrence and severity of atopic dermatitis among children aged 3–11 years, and on how it impacts their quality of life. The researchers visit the clinic 4 days a week (clinic days) for 10 weeks. The research information sheet was given to each respondent to read in English or IsiXhosa, depending on the literacy level or choice. Thereafter, verbal and written consent were obtained from the respondents before participation in the study. The parents of children diagnosed with recurrent atopic dermatitis were given the questionnaire to fill out and submit. Each questionnaire was completed in an average of 15 min – 20 min.

### Data analysis

The collected data were analysed using descriptive and inferential statistics by the Statistical Package for Social Sciences (SPSS) version 29. The children’s demographic characteristics, clinical characteristics, environmental factors and the impact of atopic dermatitis on children’s quality of life were analysed using descriptive statistics. The results were presented in a frequency table, a bar chart and a pie chart. The Kruskal–Wallis test analysed factors associated with the quality of life of children with atopic dermatitis at a 0.05 level of significance.

### Ethical considerations

Ethical approval was obtained from the Institutional Review Board (IRB), and informed consent was obtained from the respondents. Ethical clearance was obtained from the Walter Sisulu University Health Sciences Research Ethics Committee with ethics approval number 111/2024. Ethical clearance was also obtained from the Eastern Cape Health Province with the approval number EC-202408-027. Verbal and written voluntary informed consent were obtained from the respondents after they understood the study’s purpose. Respondent’s name, address and child’s hospital number were not obtained.

## Results

The demographic profile above indicates that male children (56.1%) and female children (43.9%) have atopic dermatitis. The median age of the children was 6 years (interquartile range [IQR]: 4 to 9 years). Most children were in the 4–7 age group (44.7%), followed by the 8–11 age group, while children aged 3 and younger were the least represented (19.3%). Almost all of them were of black ethnicity (99.1%). Most (86.7%) reside in rural settings around Mthatha, and all reside in formal houses. The median number of people per household was 6 (*n* = 6). The children lived an average distance of 6 km from the nearest healthcare facility.

The parents or guardians of 114 children with atopic dermatitis reported that atopic dermatitis affected various body parts, with the face affected in 38 (33.3%) of the children, hands in 27 (23.7%), feet in 23 (20.2%), legs in 11 (9.6%), upper body in 8 (7.0%), back of upper body in 5 (4.4%), entire body in 4 (3.5%) and head in 1 (0.9%) child. Atopic dermatitis affected more than two body parts in two children.

The symptoms of atopic dermatitis were first noticed at a median age of 6 months, and the children had lived with the condition for a median duration of 66 months (or 6 years). The study participants reported recurrent atopic dermatitis in 23 (20.2%) children after more than 6 months. Additionally, 31 (27.2%) of participants had immediate family members diagnosed with atopic dermatitis, 31 (27.2%) had family members diagnosed with asthma, and 24 (21.1%) had family members diagnosed with allergies.

Mild atopic dermatitis was the most common type (64.9%, *n* = 74), as recorded by the dermatologist in the children’s file, followed by moderate dermatitis (32.5%, *n* = 37). Only three (2.6%, *n* = 3) children suffered from severe atopic dermatitis.

Table 1 reveals that having pets at home (81.6%), use of regular soap (98.2%), winter season (53.9%) and lack of education on the management of atopic dermatitis (98.2%) were associated with the development, recurrence and severity of atopic dermatitis in the study population.

**TABLE 1:** Environmental factors associated with atopic dermatitis.

Variables of interest	Category	*n*	%	Median	IQR
Have pets at home	Yes	93	81.6	-	-
	No	21	18.4	-	-
Type of pet at home, *n* = 93	Cat	34	36.6	-	-
	Dog	59	63.4	-	-
The child is exposed to second-hand smoke	Yes	16	14.0	-	-
	No	98	86.0	-	-
The primary source of cooking	Electricity	102	89.5	-	-
	Gas	1	0.9	-	-
	Wood	11	9.6	-	-
The primary source of heating	Electricity	101	88.6	-	-
	Gas	1	0.9	-	-
	Wood	12	10.5	-	-
Primary source of water	Borehole	2	1.8	-	-
	Piped water	16	14.0	-	-
	River	51	44.7	-	-
	Tank	45	39.5	-	-
Have access to indoor plumbing	Yes	16	14.0	-	-
	No	98	86.0	-	-
The child was breastfed	Yes	85	74.6	-	-
	No	29	25.4	-	-
The child has known food allergies	Yes	6	5.3	-	-
	No	108	94.7	-	-
Named food allergies, *n* = 6	Cow milk	1	16.7	-	-
	Diary	2	33.3	-	-
	Eggs	1	16.7	-	-
	Peanuts	1	16.7	-	-
	Sour milk	1	16.7	-	-
Child’s skin condition worsens during a specific season	Yes	76	66.7	-	-
	No	38	33.3	-	-
Season when a child’s skin condition worsens, *n* = 76	Autumn	2	2.6	-	-
	Spring	10	13.2	-	-
	Summer	23	30.3	-	-
	Winter	41	53.9	-	-
Industrial activity taking place near the residence	Yes	0	0.0	-	-
	No	114	100.0	-	-
Air quality in the area	Excellent	1	0.9	-	-
	Good	83	72.8	-	-
	Fair	30	26.3	-	-
Frequency of child bathing	Daily	85	74.6	-	-
	Every other day	26	22.8	-	-
	2–3 times a week	3	2.6	-	-
Type of soap/cleanser used for child bathing	Mild/Unscented soap	1	0.9	-	-
	Regular soap	112	98.2	-	-
	Specialised for sensitive skin	1	0.9	-	-
Had received education on managing atopic dermatitis	Yes	2	1.8	-	-
	No	112	98.2	-	-
Breastfeeding duration (months)	-	-	-	12	6–12
Age when solid foods were introduced (months)	-	-	-	3	3–3

IQR, interquartile range.

The median impact score was one (*n* = 1) for itching or scratching, school, sleep and treatment domains, indicating that having atopic dermatitis resulted in some degree of discomfort as it caused itching or scratching, had a minor impact on schooling, affected sleep and treating the condition affected the quality of life of the children. The median score was zero (*n* = 0) for embarrassment, friendships, clothing, playing, sporting activities and teasing or bullying, implying that atopic dermatitis did not negatively affect these areas (Table 2).

**TABLE 2:** Impact of atopic dermatitis on children’s quality of life (4–11 years).

Quality of life variables	Median	IQR
Itching/scratching	1	1–1
Embarrassment	0	0–0
Friendships	0	0–0
Clothing	0	0–0
Playing	0	0–0
Sporting activities	0	0–0
School	1	0–1
Teasing/bullying	0	0–0
Sleep	1	0–1
Treatment	1	0–1
Total CDLQI	4	2–5

IQR, interquartile range; CDLQI, Children’s Dermatology Life Quality Index.

The results show that the overall quality of life of children with atopic dermatitis, as assessed by the CDLQI, was only weakly affected (median score of 4). Aspects of the CDLQI that contributed to the negative quality of life include itching or scratching, school, sleep and treatment domain (Table 2).

Categorically, atopic dermatitis had a weak effect on the quality of life of the majority (65.2%, *n* = 60) of the children, a moderate impact on 10 (10.9%) and a strong effect on the quality of life of two (2; 2.2%). No effect was reported by 20 (21.7%) of the participants (Figure 2).

The results in Table 3 show that only the severity of atopic dermatitis significantly affected the quality of life of children with the condition. Children with severe disease forms had significantly higher CDLQI scores (median: 11.5, IQR: 9–14) than those with moderate (median: 5, IQR: 4–6) or mild (median: 3, IQR: 1–4) forms. The degree of effect of atopic dermatitis on quality of life was not influenced by gender, age group or body part affected (*p* > 0.05).

**TABLE 3:** Factors associated with the quality of life of children with atopic dermatitis.

Variables of interest	Categories	CDLQI		Mann–Whitney *p*-value
		Median	(25th – 75th)	
Gender	Female	4.0	1.5–5.5	0.5670
	Male	3.0	2.0–5.0	
Age groups (years)	4–7	4.0	2.0–5.0	0.8990
	8–11	3.0	2.0–5.0	-
Severity of a child’s atopic dermatitis	Severe	11.5	9.0–14.0	< 0.0001†
	Moderate	5.0	4.0–6.0	
	Mild	3.0	1.0–4.0	
Face	Yes	4.0	3.0–6.0	0.1900
Hands	Yes	3.0	2.0–5.0	0.4030
Back of upper body	Yes	4.5	1.5–7.5	0.6910
Feet	Yes	2.5	0.0–5.0	0.2790
Full body	Yes	7.0	2.5–11.5	0.2050
Head	Yes	0.0	0.0–0.0	0.1520
Legs	Yes	3.0	3.0–4.0	0.9890
Upper body	Yes	3.0	1.0–4.0	0.5320

CDLQI, Children’s Dermatology Life Quality Index.

† , Kruskal–Wallis.

## Discussion

Atopic dermatitis is a typical skin lesion from infancy to childhood, exacerbated by environmental factors. In the current study, both male and female children were affected in a similar proportion. The age distribution of the children is similar to that reported in an Ethiopian study conducted by Kelbore et al. (2024). Also, about half of the study population was ages 4–7, which is similar to the age range reported by Ražnatović Đurović et al. (2019).

### Clinical characteristics of atopic dermatitis among children 3 to 11 years of age

Mild atopic dermatitis was the most common among the children (65%) in varied degrees (Figure 1). Findings show that severe cases of AD are rare, as only about 3% of the children presented with a severe case. This finding is inconsistent with studies that reported moderate to severe cases of atopic dermatitis among children (Kelbore et al. 2024; Na et al. 2019). The low percentage of severe cases may be related to the study’s rural setting and possibly to less exposure to urban pollutants. Also, the limited number of severe cases might reflect the success of early interventions or under-reporting of more intense symptoms. These findings suggest that early, proactive treatment could mitigate severity, though future research should investigate the long-term effects in more severely affected cases.

**FIGURE 1:**
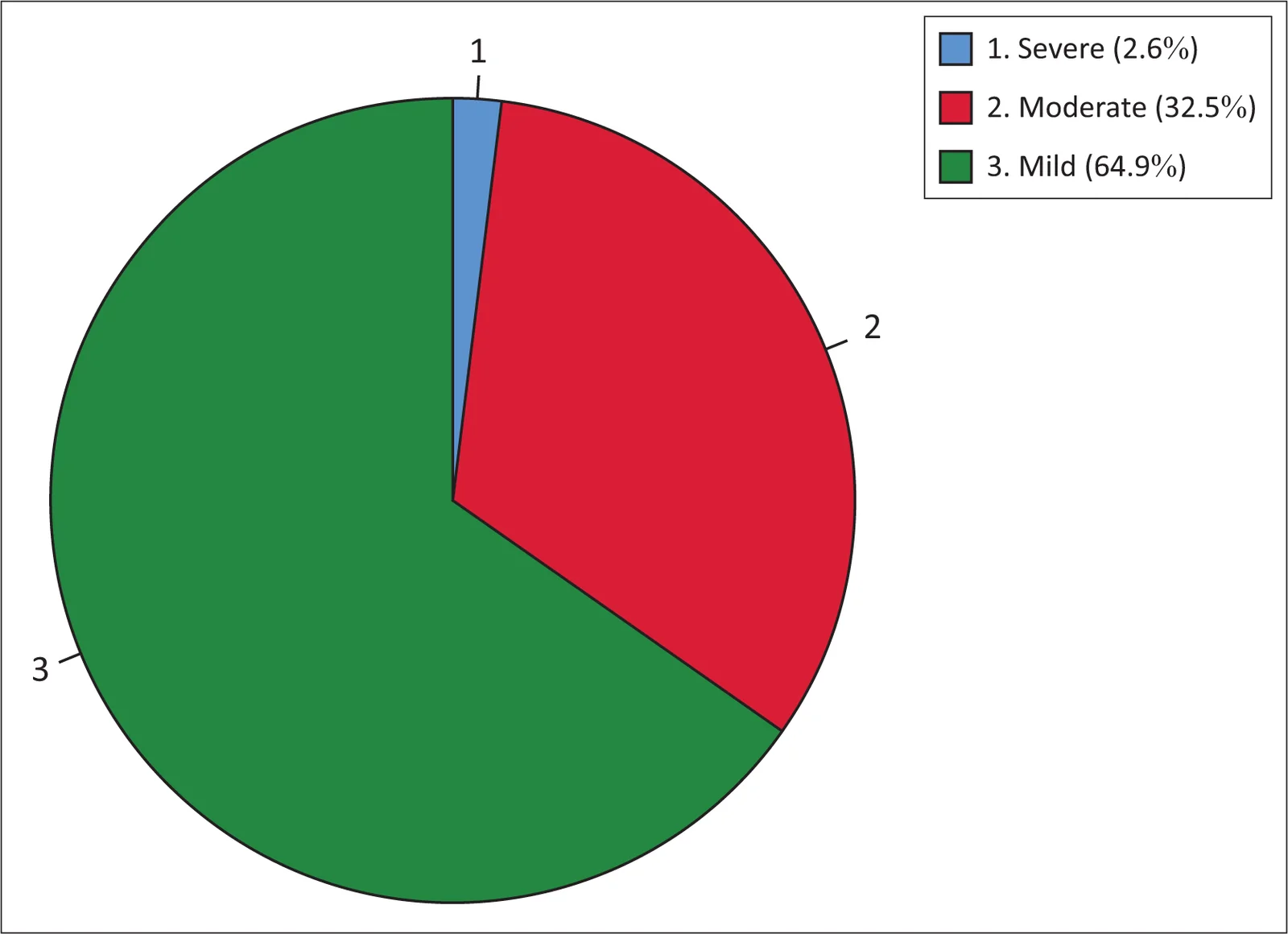
Severity of atopic dermatitis among 114 children aged 3–11 years.

The finding is inconsistent with 68% moderate to severe cases reported in a cross-sectional study in Ethiopia focusing on the clinical severity of AD among children (Kelbore et al. 2024). The Ethiopian study was multicentre, involving 461 children, whereas our study was single-centre, involving 114 children. This significant difference may influence the purported report about the severity of the condition in the study settings. However, the similarity between the two studies lies in the constant fact that children are primarily affected in their faces, hands and feet (Neri et al. 2023). In this study, the face was the most frequently impacted region (Table 4), which may influence self-consciousness and social interactions over time.

**TABLE 4:** Clinical characteristics of atopic dermatitis among children 3 to 11 years of age.

Variables of interest	Yes		No		Median	IQR
	*n*	%	*n*	%		
**Body parts affected by atopic dermatitis**						
Face	38	33.3	76	66.7	-	-
Hands	27	23.7	87	76.3	-	-
Back of upper body	5	4.4	109	95.6	-	-
Feet	23	20.2	91	79.8	-	-
Full body	4	3.5	110	96.5	-	-
Head	1	0.9	113	99.1	-	-
Legs	11	9.6	103	90.4	-	-
Upper body	8	7.0	106	93.0	-	-
Atopic dermatitis is recurrent after more than 6 months	23	20.2	91	79.8	-	-
Immediate family member(s) had been diagnosed with atopic dermatitis	31	27.2	83	72.8	-	-
Immediate family member(s) had been diagnosed with asthma	31	27.2	83	72.8	-	-
Immediate family member(s) had been diagnosed with allergies	24	21.1	90	78.9	-	-
Age when first symptoms of atopic dermatitis were noticed (months)	-	-	-	-	6	3–6
Duration of living with atopic dermatitis (months)	-	-	-	-	66	42–93
Duration of living with atopic dermatitis (years)	-	-	-	-	6	4–8

IQR, interquartile range.

The symptoms first appeared at a median age of 6 months, and children lived with the condition for approximately 6 years, emphasising the chronic nature of the disease in this population. This observation supports inferences from extant studies, which underlined that atopic dermatitis typically begins in infancy and can persist through childhood, significantly impacting children’s well-being over prolonged periods (Koszorú et al. 2019; Sandeep, Wade & Stuart 2018; Staab et al. 2000). According to the findings, parents lacked formal knowledge of managing atopic dermatitis. Critically, these warrants question the parents’ actions, as treatment was not sought within 6 years of a healthcare facility being available and within 6 km (Table 5). A further study is needed to examine the parents’ propensity to utilise the available health facilities in the locality. Considering the role of education in mitigating the development, recurrence and severity of atopic dermatitis, Muzzolon et al. (2021) conducted a clinical trial to evaluate the effect of educational intervention (EI) on the severity of AD and its impact on children’s quality of life. The study concluded that EI decreases AD severity and improves children’s quality of life. Similarly, Staab et al. (2000) emphasised the importance of family education on management and coping strategies for children with atopic dermatitis.

**TABLE 5:** Demographic characteristics of children with atopic dermatitis.

Demographic variables	Category	*N* = 114		Median	IQR
		*n*	%		
Gender	Female	50	43.9	-	-
	Male	64	56.1	-	-
	Total	114	100.0	-	--
Age (years)	≤ 3	22	19.3	-	-
	4–7	51	44.7	-	-
	8–11	41	36.0	-	-
	-	-	-	6	4–9
Ethnicity	Black people	113	99.1	-	-
	Indian people	1	0.9	-	-
Area of residence	Rural	98	86.7	-	-
	Urban	15	13.3	-	-
Type of housing	Formal	114	100.0	-	-
Number of people living in the household	-	-	-	6	4–7
Nearest healthcare facility from home (km)	-	-	-	6	4–8

IQR, interquartile range.

Furthermore, it could be argued that AD was mildly experienced among the children, and parents do not consider any reason for the hospital visit for this condition. A home remedy may treat the ailment rather than a clinic visit, which can take up much of a parent’s time and resources. In addition, information on parents’ level of education and work schedule was not obtained, which may corroborate the findings in future studies. Some studies in Africa and Europe emphasised that atopic dermatitis often presents early and varies in severity. For instance, Kelbore et al. (2024) reported AD development within the first 2 years of life, which is believed to be associated with the level of IgE. Atopic dermatitis development occurs much earlier in the current study. Also, a few studies have associated the early development of AD with more complicated cases in the future (Guttman-Yassky, Renert-Yuval & Brunner 2025; Sandeep et al. 2018).

Moreover, apart from atopic dermatitis, immediate family members were also diagnosed with asthma and allergies. This finding corroborates (Guttman-Yassky et al. 2025; Koszorú et al. 2019; Sandeep et al. 2018) the assertion that atopic dermatitis is associated with bronchial asthma, food allergies, allergic rhinitis and other allergic conditions. However, it is not clear whether atopic dermatitis can exacerbate hypersensitivity reactions in children. One suggestion is to conduct a multi-centre clinical trial and compare results. Nonetheless, some studies have reported that children who developed AD have a family or close relative who has had the disease (Koszorú et al. 2019; Na et al. 2019). This suggests that genetics and environmental factors continue to play significant roles in the development of atopic dermatitis. The environmental factors leading to the development and recurrence of chronic atopic dermatitis are an important niche area for scientific research.

### Environmental factors associated with atopic dermatitis

According to our findings, the children were reported to live in a formal house in a rural setting. We could not compare the description of the formal house in a rural setting in South Africa with rural housing arrangements elsewhere. Nonetheless, more important data were the number of people living in the house, which was reported to be six per household. In essence, overcrowding, poverty and parents’ level of education may be associated with atopic dermatitis in most settings. This assumption is in line with Jelousi et al. (2022), who reported that poor living conditions and climate change aggravated the development of atopic dermatitis. Similarly, Polaskey and Chovatiya found that social and structural determinants of health, including the environment, economy, education, access to healthcare and societal values, influence individuals’ risk of developing and treating atopic dermatitis.

Curiously, the children were breastfed for 12 months, while solid foods were introduced at 3 months. Although this study was not designed to investigate the cause and effect of inadequate breastfeeding and early complementary feeding on the development of atopic dermatitis, it raises a cynical concern about the role of adequate infant nutrition in the prevention of diseases. The World Health Organization (WHO) recommends exclusive breastfeeding for the first 6 months (Bonamonte et al. 2019). However, there is a dramatic change in this recommendation in recent studies investigating the relationship between exclusive breastfeeding and the development of atopic dermatitis. The relationship between breastfeeding and atopic dermatitis has not been well studied (Hachmeriyan et al. 2025): A systematic review and meta-analysis covering 27 studies conducted by Lin et al. (2020) found no association between AD and breastfeeding. Similarly, a recent prospective cohort study in Bulgaria reported no relationship between breastfeeding and infants’ protection against allergies (Toneva et al. 2025), while a prospective observational study aimed at evaluating whether exclusive breastfeeding should be discontinued for a child with atopic dermatitis concluded that breastfeeding should be discontinued to improve symptoms and reduce the severity of atopic dermatitis (Lin et al. 2019). Also, Wang et al. (2020) posited that exclusive breastfeeding should be discontinued to improve symptoms of atopic dermatitis and further argued that infants who are exclusively breastfed are at risk of AD due to reduced levels of vitamin D in the breast milk.

Moreover, environmental factors play a vital role in the development, recurrence and severity of atopic dermatitis. However, according to Narla and Silverberg (2020), it is challenging to specify which environmental exposures are responsible for exacerbating atopic dermatitis at any given time. Therefore, it was advised that treatment should focus on all known environmental factors contributing to the disease (Bonamonte et al. 2019) exposited on various environmental factors contributing to atopic dermatitis: On climate, the study supported the assumption that vitamin D deficiency aggravates the manifestation of atopic dermatitis. The risk of AD was reported to be lower in rural settlements than in urban settlements. The role of diet in AD development is poorly understood. However, Bonamonte et al. cited a study that argued that high consumption of fish during the prenatal period and early childhood lowers the risk of developing AD by 40%.

In this study, environmental factors contributing to AD include keeping pets, particularly dogs and/or cats, at home, using regular soap and the winter season. Seasonal variation showed that winter was the most challenging season, with over 50% of children experiencing worsening symptoms, possibly related to drier air. Limited access to treated water could expose young children in the area to allergens. Furthermore, regular soap was the most commonly used bathing product. It may contribute to skin irritation from chemicals and fragrances in regular soap, underscoring the need for public health education on gentle skin care for atopic dermatitis. Altogether, these environmental findings emphasise the role of daily living conditions and highlight actionable steps to reduce exacerbation risks.

### Impact of atopic dermatitis on children’s quality of life (4–11 years)

The CDLQI scale was used to determine the impact of atopic dermatitis on children’s quality of life. From the findings, atopic dermatitis has a weak effect on the quality of life of most of the children (65.2%) (see Figure 2). This is simply because atopic dermatitis was found to be mild in terms of the location and spread on the body part for many of the children. Only two children were severely impacted by atopic dermatitis. Statistically, atopic dermatitis has a median impact score of 1 (*n* = 1) in the itching or scratching, schooling, sleeping and treatment domains (see Table 2). This finding is consistent with a clinical review by Koszorú et al. (2019) which reported that atopic dermatitis affects children’s quality of life due to its effect on sleep, work productivity and emotional and mental health. It is also in line with Na et al. (2019). Atopic dermatitis is associated with itching, leading to frequent scratching and sleep loss. Again, Xu et al. (2019), in a study on factors influencing quality of life in children with atopic dermatitis, itching or scratching and sleep disturbance found that these factors were associated with the severity of atopic dermatitis.

**FIGURE 2:**
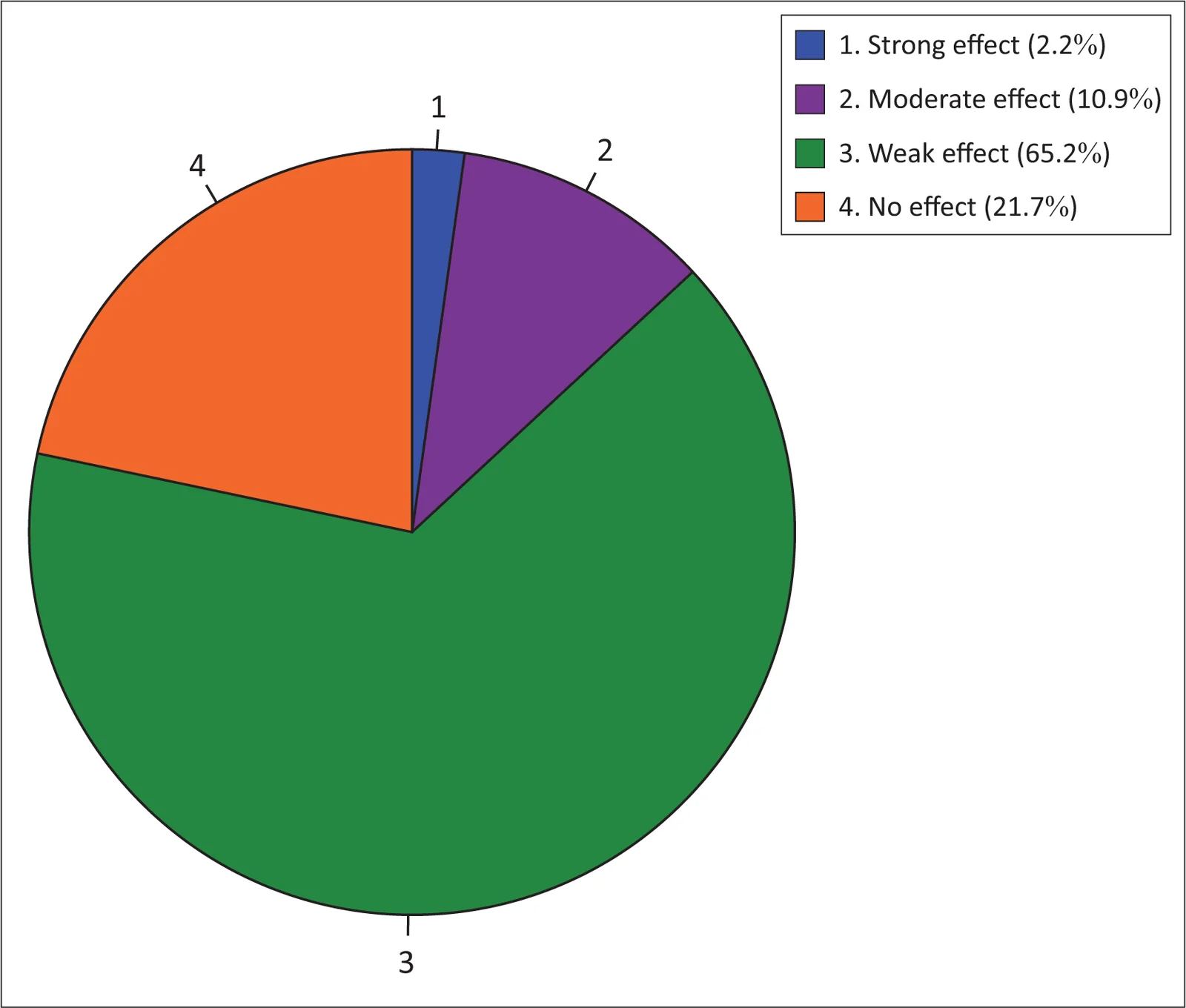
Impact of atopic dermatitis on the quality of life of children aged 4–11 years using the Children’s Dermatology Life Quality Index scale.

An Italian study on the implications of atopic dermatitis on the quality of life of young children emphasised that AD significantly caused disturbed sleep patterns with daytime drowsiness (Meltzer et al. 2020; Neri et al. 2023). Similarly, a study in Romania on AD severity using multiple assessment tools reported a correlation between atopic dermatitis severity and impact on children’s quality of life (Sur et al. 2020). Furthermore, itching may impede day care and school attendance to an extent. Also, scratching the affected area and recurrence after 6 months are concerning and may disturb children and their parents (Koszorú et al. 2019). However, the median score was zero (*n* = 0) for embarrassment, friendships, clothing, playing, sporting activities and teasing or bullying, implying that atopic dermatitis did not negatively affect these areas (see Table 2). Overall, AD did not threaten children’s survival and quality of life in the study population. Conversely, in a survey among preadolescents aged 6–11 in Italy, parents of children with AD reported bullying at school and inability to join a sports activity, which led to the victim’s absenteeism (Neri et al. 2023).

Regarding factors associated with the quality of life of children with atopic dermatitis, a computed Kruskal–Wallis test shows that only the severity of atopic dermatitis significantly affects children’s quality of life. The CDLQI score was higher in children with severe atopic dermatitis (median: 11.5, IQR: 9–14) (see Table 3), but, statistically, the quality of life of children with atopic dermatitis was not influenced by gender, age group or the body part affected. Similarly, in a 2018 cross-sectional study investigating the influence of age on the quality of life of children with atopic dermatitis in Podgorica, a negative correlation between age and the CDLQI score was observed (Ražnatović Đurović et al. 2019). Importantly, public health interventions should focus on preventing the recurrence and severity of atopic dermatitis. The local health authority can mitigate the effects of atopic dermatitis among preschoolers and school-age children through measures that include awareness campaigns on minimising indoor triggers, such as dry air from heating systems, educating families on strategies to maintain skin hydration during winter, limiting pet access to sleeping areas or offering referrals to allergists for further assessment in severe cases and addressing gaps identified in rural healthcare system.

### Limitations of the study

The study was conducted in a rural setting near Mthatha, and the inferences are not generalisable to all children with atopic dermatitis in all rural settings in South Africa. The study follows a cross-sectional research design and does not aim to determine the causes and effects of atopic dermatitis, its recurrence or its impact on children’s quality of life. Future studies will compare the impact of AD on children’s quality of life between the urban centre and the countryside. Multiple assessment scales will also be deployed to increase the study’s precision.

## Conclusion

This study discusses the demographic, clinical and environmental factors influencing AD among children aged 3 to 11 in rural areas near Mthatha, Eastern Cape, South Africa, emphasising the disease’s significant but context-specific impact on their quality of life. The findings reveal a predominance of mild AD cases, an onset predominantly during childhood and unique influences from environmental factors like pet exposure, soap use and seasonal changes. The quality of life of children who live in rural areas is primarily impacted by atopic dermatitis in areas such as schooling, sleeping and treatment modality.
